# Texture analysis of apparent diffusion coefficient maps in predicting the clinical functional outcomes of acute ischemic stroke

**DOI:** 10.3389/fneur.2023.1132318

**Published:** 2023-05-11

**Authors:** Yi Sun, Yuzhong Zhuang, Jie Zhu, Bin Song, Hao Wang

**Affiliations:** Department of Radiology, Minhang Hospital, Fudan University, Shanghai, China

**Keywords:** ischemic stroke, radiology, diffusion magnetic resonance imaging, texture analysis, prognosis

## Abstract

**Purpose:**

To investigate texture analysis (TA) based on apparent diffusion coefficient (ADC) map in predicting acute ischemic stroke (AIS) prognosis and discriminating TA features in stroke subtypes.

**Methods:**

This retrospective study included patients with AIS between January 2018 and April 2021. The patients were assigned to the favorable [modified Rankin Scale (mRS) score ≤ 2] and unfavorable (mRS score > 2) outcome groups. All patients underwent stroke subtyping according to the Trial of Org 10,172 in Acute Stroke Treatment (TOAST) classification. The TA features were extracted from infarction lesions on the ADC map. The demographic characteristics, clinical characteristics, and texture features were used to construct prediction models with recurrent neural network (RNN). The receiver operating characteristic (ROC) curves were implemented to evaluate the performance of the predictive models.

**Results:**

A total of 1,003 patients (682 male; mean age 65.90 ± 12.44) with AIS having documented the 90-day mRS score were identified, including 840 with favorable outcomes. In the validation set, the area under the curve (AUC) of the predictive model using only clinical characteristics achieved an AUC of 0.56, texture model 0.77, the model combining both clinical and texture features showed better with an AUC of 0.78. The texture feature profiles differed between large artery atherosclerosis (LAA) and small artery occlusion (SAO) subtypes (all *p* < 0.05). The AUC of combined prediction models for LAA and SAO subtypes was 0.80 and 0.81.

**Conclusion:**

Texture analysis based on ADC map could be useful as an adjunctive tool for predicting ischemic stroke prognosis.

## Introduction

Stroke remains the second-leading cause of death and the third-leading cause of death and disability combined globally (1). The burden of stroke has increased substantially over the past few decades due to an increasing and aging population as well as the increased prevalence of modifiable stroke risk factors, particularly in low-and middle-income countries (2). More than 12 million people worldwide suffer a stroke each year, and approximately 70–80% of stroke cases are attributed to ischemic stroke (3, 4). The distribution of ischemic stroke subtypes varies among different racial or ethnic groups (5). Since accurate clinical strategies can improve the outcomes in patients with acute ischemic stroke (AIS), the obtainability of robust and validated prognostic biomarkers is essential to optimize early individualized therapy and rehabilitation strategies.

Texture analysis (TA) is an effective quantitative image analysis tool to explore the microstructural changes that cannot be explored by humans visually. TA defines the measure of voxel intensities, voxel inter-relationships, and the gray-level patterns in the image. TA has been successfully used for characterizing multiple sclerosis (6–8), small vessel disease (9), and dementia with Lewy bodies (10). It has been demonstrated that TA is an effective tool for image analysis. In the field of ischemic stroke, TA based on magnetic resonance imaging (MRI) has been reported to be applied to the early identification of ischemic lesions (11), stroke severity classification (12), post-stroke cognitive impairment (13) and detecting the effects of stroke therapy (14).

Diffusion-weighted imaging (DWI) is the most important and commonly used part of routine clinical stroke neuroimaging protocols as it facilitates the identification of stroke lesions. The apparent diffusion coefficient (ADC) yielded by DWI is sensitive to the initial cell swelling of a cytotoxic edema. ADC maps have been found to reveal the early indications and progression of cerebral ischemic infarction (15). Each voxel intensity of ADC maps is determined partly by intracellular water and partly by extracellular water. The changes in the distribution of extracellular water entering cells after infarction may possibly be reflected in the texture features. Previous studies demonstrated that the texture features based on DWI and ADC maps could evaluate ischemic stroke severity (12, 16). ADC changes in motor structures have also been shown to be predictors of acute stroke outcomes (17). A recent study reported that radiomics features based on DWI and ADC could predict stroke outcomes (18). However, the information on whether the texture features of infarct lesions correlate with the prognosis of AIS is limited.

We hypothesized that the ADC-based texture features might differ between patients with AIS having favorable and unfavorable clinical outcomes. Thus, this study aimed to explore the role of texture features in predicting AIS prognosis. We further investigated the characteristics difference of texture features in different stroke subtypes.

## Materials and methods

### Study population

**Figure 1:**
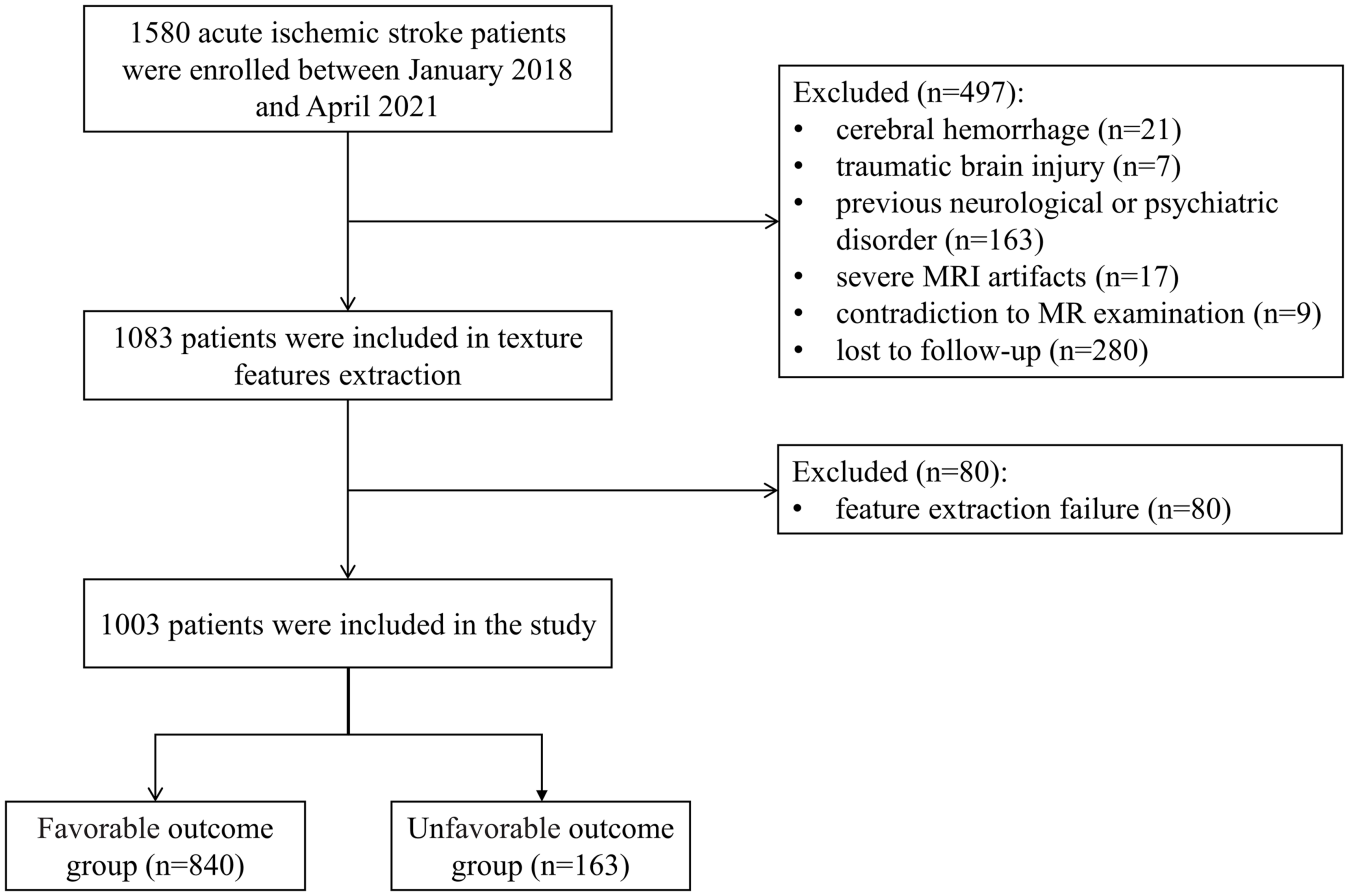
Flowchart of case selection for analysis.

Between January 2018 and April 2021, all participants, aged >18 years, presenting to our stroke center with signs and symptoms of AIS were enrolled in this retrospective study. The study had approval from the institutional ethics committee of our hospital (approval number: 2022–013-01 K). Patients with confirmed acute DWI lesions on brain MRI scans performed within 72 h of symptom onset were included in this analysis. Of 1,580 participants, we excluded participants with cerebral hemorrhage (*n* = 21), traumatic brain injury (*n* = 7), previous neurological or psychiatric disorder (*n* = 163), severe MRI artifacts (*n* = 17), contradiction to MR examination (*n* = 9), feature extraction failure (*n* = 80), or loss to follow-up (*n* = 280). The flowchart is shown in Figure 1. The requirement for informed consent was waived because of the retrospective nature of the study.

### Clinical variables

Age, sex, National Institutes of Health Stroke Scale (NIHSS) score, antecedent hypertension, diabetes mellitus, hyperlipidemia, atrial fibrillation, tobacco or alcohol use, low-density lipoprotein cholesterol (LDL-C), and discharge medications were abstracted from the medical record. The AIS subtypes were assigned according to the Trial of Org 10,172 in Acute Stroke Treatment (TOAST) classification (19).

### Clinical outcome assessment

Patients or their caregivers were interviewed in person or by telephone at 90 days after stroke to assess the functional outcomes using the modified Rankin Scale (mRS) score. The patients were assigned to the favorable (mRS score ≤ 2) and unfavorable (mRS score > 2) outcome groups (20).

### MRI examination

All MRI examinations were performed on a 1.5-T MRI scanner (GE Healthcare, WI, United States) or a 3.0-T MRI scanner (United Imaging Healthcare, Shanghai, China). The scan parameters for the 1.5-T scanner were axial DWI based on a single-shot echo planar imaging (SSEPI) sequence, with repetition time (TR)/echo time (TE) = 3,203 ms/83.9 ms, slice thickness/gap = 5 mm/1.5 mm, FOV = 240 × 240 mm^2^, *b* values = 0 and 1,000 s/mm^2^, and matrix = 96 × 96. The scan parameters for the 3.0-T scanner were axial DWI based on the SSEPI sequence, with TR/TE = 2,800 ms/75.4 ms, slice thickness/gap = 5 mm/1.5 mm, FOV = 230 × 220 mm^2^, *b* values = 0 and 1,000 s/mm^2^, and matrix = 128 × 128.

### Segmentation of infarction lesions

**Figure 2:**
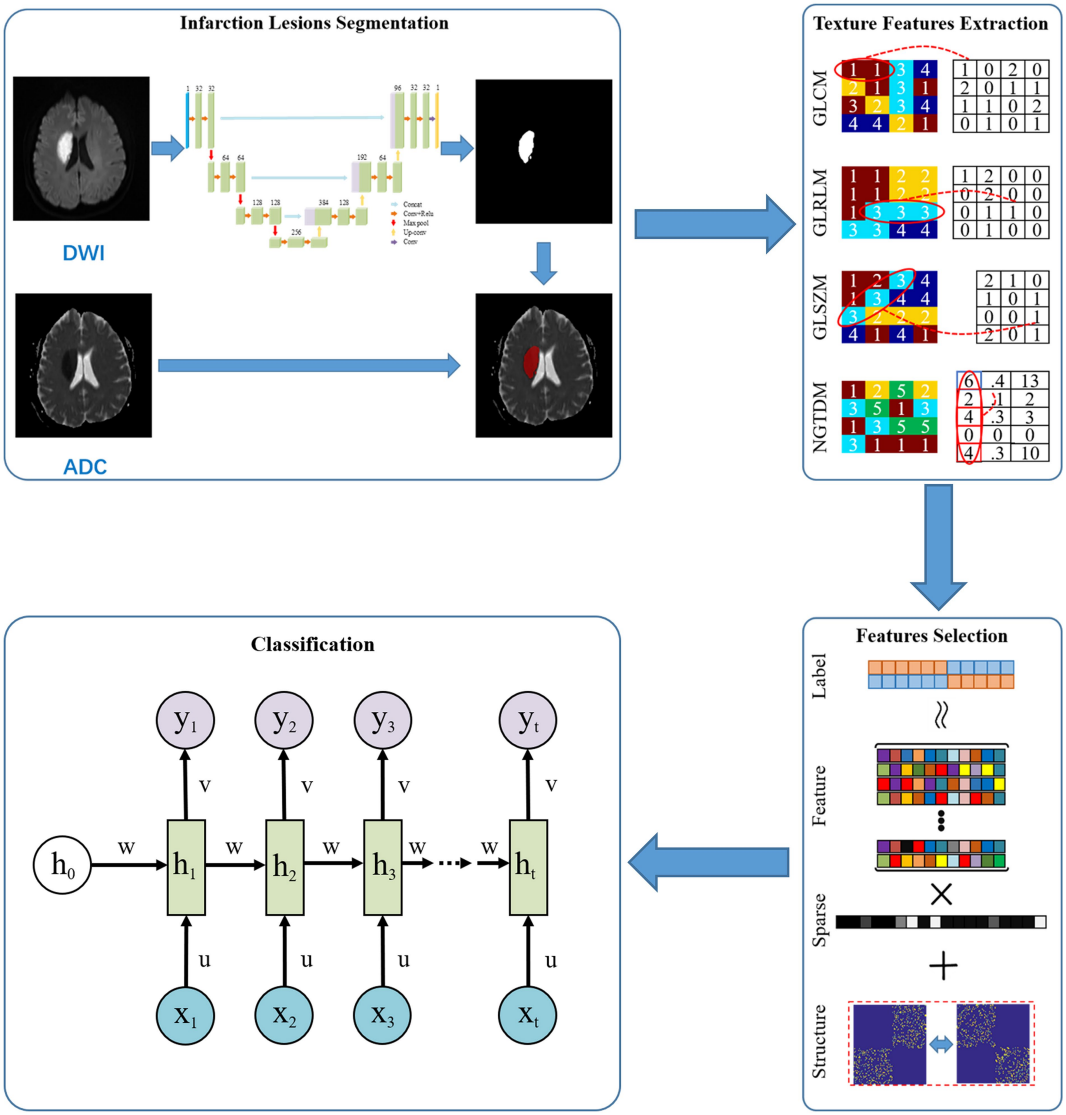
Pipeline of texture analysis for clinical outcome prediction.

A total of 100 patients were randomly selected for manual segmentation by ITK-SNAP 3.8.^1^ The volume of interest (VOI) was sketched by slice-by-slice stacking on DWI. The 100 VOIs were brought into a 2D U-Net network for automatic segmentation (21). Then, the trained segmentation network model was used to segment the remaining cases. For automatic segmentation assessment, 100 random cases from the testing set were chosen to calculated the DICE coefficient. Image intensity parameters were normalized to 0–1 using window width and window level before the process of automatic image segmentation. The region of VOI on DWI was copied to the corresponding ADC maps. The mask matrix based on DWI and ADC maps was used for further texture extraction. The framework of the proposed method is given in Figure 2.

### Texture features extraction and analysis

Texture features extraction were performed on the MATLAB 2019a (The MathWorks Inc., Natick, MA, USA). Based on the extraction results of texture features and clinical characteristics, we use sparse representation for feature selection to reduce the redundant information in features and improve classification model accuracy. Specifically, we use sample features for sparse representation in sample labeling, so the highest label correlated feature subsets are selected. Meanwhile, the sparsity constraints on the model effectively removed the correlation and redundancy in the features of the feature subsets. Following is the model: $\hat{w}=\underset{w}{\arg\min}∥l-Fw{∥}_{2}^{2}+\eta ∥w{∥}_{0}$ (22). $l\in R^{m}$ being the training sample label, m being the size of the training sample, $F={\left[f_{1},f_{2}\cdots f_{m}\right]}^{T}\in R^{m\times 2K}$ being feature set of training sample, ? being the sparse representation parameter, the absolute value of factors in sparse representation coefficient, w is the importance of the features. When w is computed, key features can be selected by simply comparing the thresholds.

The following features were extracted: eight gray-level co-occurrence matrix (GLCM) features, thirteen gray-level run-length matrix (GLRLM) features, thirteen gray-level size zone matrix (GLSZM) features, and five neighborhood gray-tone difference matrix (NGTDM) features. Additional information on TA features is provided in Supplementary Table S1.

### Model classification and data distribution

Based on the selected feature subsets, we established our classification prediction models based on recurrent neural network (RNN) (23). Weighted cross-entropy loss function was used to optimize the network. In network training, we used an Adam optimizer with a learning rate of 0.0001 and a batch size of 10.

The patients were randomly divided into the training and validation sets by stratified sampling. Due to the imbalance of sample number in our study, we used under-sampling method to build the dataset. The unfavorable-outcome group was randomly divided into training and validation sets at the ratio of 2:1. Then, the patients in the favorable-outcome group were randomly assorted into the training set 1.5 times the number in the training set with an unfavorable outcome. The remaining patients were included in the validation set to validate the reliability and robustness of the models.

### Statistical analysis

The continuous variables with normal distribution were reported as mean ± standard deviation, non-normally distributed variables as median (interquartile range), and classification variables as frequency (%). We used the independent-samples *t-*test or Mann–Whitney *U* test for continuous variables and the chi-square for categorical dependent variables between favorable-outcome and unfavorable-outcome groups, as appropriate. The receiver operating characteristic (ROC) curve was generated to evaluate the performances of the predictive models. All statistical analyses were carried out using SPSS (version 26.0, SPSS Inc., Chicago, IL, United States). All the reported *p* values were based on the two-tailed tests, and *p* values less than 0.05 indicated a statistically significant difference.

## Results

### Demographic and clinical characteristics

We included 1,003 patients with AIS having a 90-day mRS score available for this analysis. Of these, 682 (68.0%) were men, and the mean age was 65.90 years (SD 12.44 years). Table 1 summarizes the demographic and clinical features of the study population and comparisons between the groups. In reproducibility analysis, the manually drawn results and the network’s automatic segmentation exhibited excellent agreement (DICE coefficient = 0.886).

**Table 1:** Demographic and clinical characteristics of AIS patients with favorable and unfavorable outcome.

Characteristics	Functional outcome		*p-*value
	Favorable outcome (*n* = 840)	Unfavorable outcome (*n* = 163)	
Age, y	66.02 ± 12.21	65.33 ± 13.57	0.516
Men, *n* (%)	578 (68.8%)	104 (63.8%)	0.210
Smoking, *n* (%)	318 (37.9%)	60 (36.8%)	0.801
Drinking, *n* (%)	114 (13.6%)	20 (12.3%)	0.655
Hypertension, *n* (%)	563 (67.0%)	113 (69.3%)	0.566
Hyperlipidemia, *n* (%)	229 (27.3%)	48 (29.4%)	0.568
Diabetes Mellitus, *n* (%)	292 (34.8%)	58 (35.6%)	0.841
Atrial fibrillation, *n* (%)	92 (11.0%)	21 (12.9%)	0.475
Stroke subtype (TOAST), *n* (%)			**0.012**
LAA	445 (53.0%)	99 (60.7%)	
CE	66 (7.9%)	14 (8.6%)	
SAO	262 (31.2%)	36 (22.1%)	
Other	7 (0.8%)	6 (3.7%)	
Undetermined	60 (7.1%)	8 (4.9%)	
Discharge statin, *n* (%)	526 (62.6%)	108 (66.3%)	0.378
Discharge antiplatelet, *n* (%)	759 (90.4%)	138 (84.7%)	**0.030**
Discharge anticoagulant, *n* (%)	39 (4.6%)	6 (3.7%)	0.587
LDL-C mmol/L	3.01 ± 0.93	3.09 ± 1.13	0.314
Admission NIHSS score	3 (1–4)	4 (2–7)	**<0.001**
Stroke volume, ml	1.68 (0.70–7.12)	2.35 (0.78–13.41)	**<0.001**

LAA, large artery atherosclerosis; CE, cardioembolism; SAO, small artery occlusion; HbA1c, glycosylated hemoglobin; LDL-C, low-density lipoprotein cholesterol; NIHSS, National Institutes of Health Stroke Scale. The bold values indicate the value of p less than 0.05.

### Functional outcomes in the entire cohort

Of 1,003 patients, 840 (83.7%) showed functional independence (mRS ≤ 2) at 90 days. Further, 163 participants (16.3%) had unfavorable outcomes (74 patients with mRS = 3, 47 with mRS = 4, 28 with mRS = 5, and 14 with mRS = 6). Strokes with favorable outcomes had a lower admission NIHSS score and smaller stroke volume (both *p* < 0.001). The stroke subtype showed a significant difference between the two groups (*p* = 0.012). The patients with unfavorable outcomes were less likely to receive antiplatelet therapy after discharge (*p* = 0.030) (Table 1).

Eleven texture features, including five GLRLM features, five GLSZM features, and one NGTDM feature demonstrated statistically significant differences. The information of the 11 features is provided in Table 2.

**Table 2:** Texture features analysis (*p* < 0.05) in AIS patients with and without favorable outcome.

Method	Texture features	*p-*value
GLRLM (gray-level run-length matrix)	Short run emphasis (SRE)	0.008
	Long run emphasis (LRE)	0.006
	Gray-level nonuniformity (GLN)	<0.001
	Run-length nonuniformity (RLN)	<0.001
	Run percentage (RP)	0.007
GLSZM (gray-level size zone matrix)	Gray-level nonuniformity (GLN)	<0.001
	Zone-size nonuniformity (ZSN)	<0.001
	Zone percentage (ZP)	0.003
	High gray-level zone emphasis (HGZE)	0.043
	Large zone low gray-level emphasis (LZLGE)	0.004
NGTDM (neighborhood gray-tone difference matrix)	Busyness	<0.001

**Figure 3:**
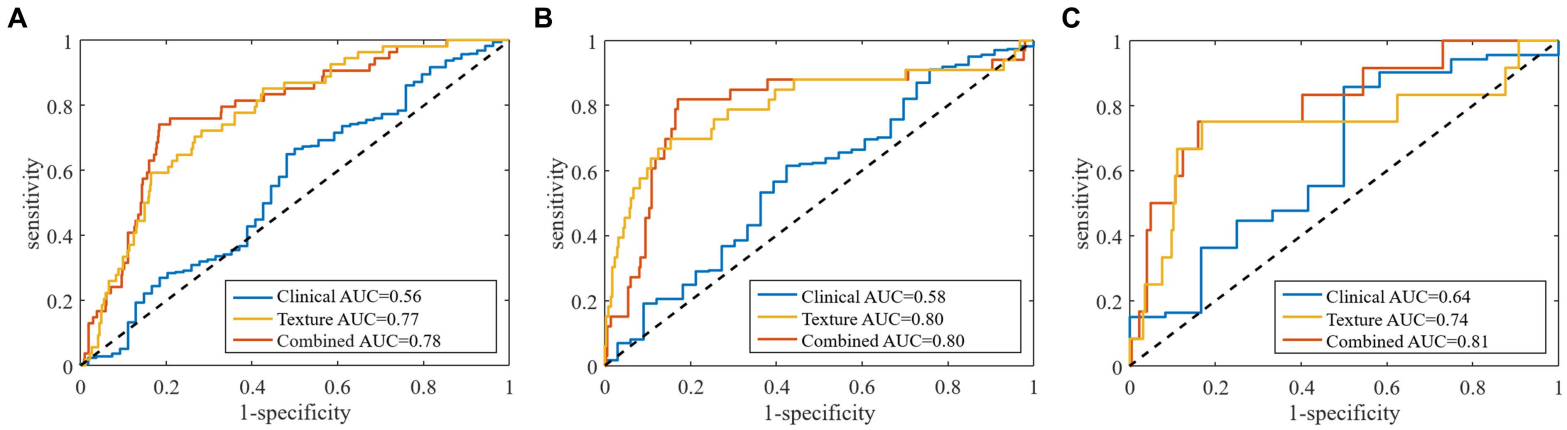
Receiver operator characteristic curves for **(A)** total stroke, **(B)** large artery atherosclerosis type, and **(C)** small artery occlusion type by clinical characteristics, texture features and combined models in predicting of stroke outcomes.

Based on RNN, all the clinical models and texture models were constructed, the clinical characteristics and texture feature selection was shown in the Supplementary Table S2. According to the above clinical characteristics and texture features, the combined model was constructed. In the validation set, the area under the ROC curve (AUC) of the combined prediction model was 0.78, and the accuracy, sensitivity, and specificity were 0.81, 0.74, and 0.82, respectively (Table 3). The AUC of the texture model was 0.77. The model using only the clinical characteristics achieved a low AUC of 0.56 in the validation cohort (Figure 3A).

**Table 3:** The performance of the prediction models.

	Total AIS			LAA subtype			SAO subtype		
	Clinical	Texture	Combined	Clinical	Texture	Combined	Clinical	Texture	Combined
AUC	0.56	0.77	0.78	0.58	0.80	0.80	0.64	0.74	0.81
Accuracy	0.69	0.78	0.81	0.61	0.82	0.83	0.79	0.84	0.84
Sensitivity	0.41	0.61	0.74	0.55	0.70	0.79	0.50	0.67	0.75
Specificity	0.71	0.79	0.82	0.61	0.83	0.83	0.81	0.85	0.84

AUC, area under curve; AIS, acute ischemic stroke; LAA, large artery atherosclerosis; CE, cardioembolism; SAO, small artery occlusion.

### Functional outcomes in the large artery atherosclerosis type

A summary of the demographic and clinical features of the patients with large artery atherosclerosis (LAA) having different clinical outcomes is shown in Table 4. The patients resulting in favorable 90-day outcomes had a lower NIHSS score at admission and smaller stroke volume than those with unfavorable outcomes (both *p* < 0.001). The proportion of statin after discharge in the unfavorable-outcome group was higher than that in the favorable group (*p* = 0.045).

**Table 4:** Demographic and clinical characteristics in LAA and SAO strokes with favorable and unfavorable outcome.

Characteristics	LAA		*p-*value	SAO		*p-*value
	Favorable outcome (*n* = 445)	Unfavorable outcome (*n* = 99)		Favorable outcome (*n* = 262)	Unfavorable outcome (*n* = 36)	
Age, y	66.17 ± 12.19	65.21 ± 12.59	0.483	64.15 ± 12.03	66.06 ± 13.88	0.383
Men, *n* (%)	295 (66.3%)	68 (68.7%)	0.647	194 (74.0%)	22 (61.1%)	0.103
Smoking, *n* (%)	154 (34.6%)	39 (39.4%)	0.368	111 (42.4%)	13 (36.1%)	0.475
Drinking, *n* (%)	59 (13.3%)	15 (15.2%)	0.619	44 (16.8%)	4 (11.1%)	0.553
Hypertension, *n* (%)	292 (65.6%)	74 (74.7%)	0.080	180 (68.7%)	20 (55.6%)	0.115
Hyperlipidemia, *n* (%)	125 (28.1%)	34 (34.3%)	0.216	65 (24.8%)	8 (22.2%)	0.735
Diabetes Mellitus, *n* (%)	140 (31.5%)	35 (35.4%)	0.453	99 (37.8%)	14 (38.9%)	0.898
Atrial fibrillation, *n* (%)	36 (8.1%)	11 (11.1%)	0.333	5 (1.9%)	1 (2.8%)	0.740
Discharge statin, *n* (%)	276 (62.0%)	72 (72.7%)	**0.045**	164 (62.6%)	21 (58.3%)	0.621
Discharge antiplatelet, *n* (%)	411 (92.4%)	86 (86.9%)	0.79	248 (94.7%)	33 (91.7%)	0.732
Discharge anticoagulant, *n* (%)	17 (3.8%)	3 (3.0%)	0.934	2 (0.8%)	0 (0.0%)	1.000
LDL-C, mmol/L	3.05 ± 0.96	3.15 ± 1.24	0.337	2.99 ± 0.86	3.01 ± 0.90	0.895
Admission NIHSS score	3 (2–5)	4 (3–8)	**<0.001**	2 (1–3)	2 (1–3)	0.343
Stroke volume, ml	3.68 (1.41–13.40)	6.19 (1.59–15.02)	**<0.001**	0.56 (0.30–0.93)	0.41 (0.27–0.64)	0.198

LAA, large artery atherosclerosis; SAO, small artery occlusion; HbA1c, glycosylated hemoglobin; LDL-C, low-density lipoprotein cholesterol; NIHSS, National Institutes of Health Stroke Scale. The bold values indicate the value of p less than 0.05.

Thirteen texture features, including five GLRLM features, seven GLSZM features, and one NGTDM feature demonstrated statistically significant differences. The information of the 13 features is provided in Table 5.

**Table 5:** Texture features analysis (*p* < 0.05) in the large artery atherosclerosis type between favorable-outcome and unfavorable-outcome groups.

Method	Texture features	*p-*value
GLRLM (gray-level run-length matrix)	Short run emphasis (SRE)	0.041
	Long run emphasis (LRE)	0.049
	Gray-level nonuniformity (GLN)	<0.001
	Run-length nonuniformity (RLN)	<0.001
	Run percentage (RP)	0.040
GLSZM (gray-level size zone matrix)	Large zone emphasis (LZE)	<0.001
	Gray-level nonuniformity (GLN)	<0.001
	Zone-size nonuniformity (ZSN)	<0.001
	Zone percentage (ZP)	0.005
	High gray-level zone emphasis (HGZE)	0.022
	Large zone low gray-level emphasis (LZLGE)	0.008
	Large zone high gray-level emphasis (LZHGE)	<0.001
NGTDM (neighborhood gray-tone difference matrix)	Busyness	<0.001

The clinical model, including stroke volume, NIHSS score, discharge statin, discharge anticoagulant and LDL-C, exhibited an AUC of 0.58 with accuracy, sensitivity and specificity of 0.61, 0.55 and 0.61, respectively, in the validation cohort. The effectiveness of clinical model was lower than the texture model (AUC: 0.80) and the combined model (AUC: 0.80) (Table 3 and Figure 3B).

### Functional outcomes in the small artery occlusion type

A total of 298 patients, including 262 with favorable outcomes and 36 with unfavorable outcomes, were classified into the small artery occlusion (SAO) type. No significant differences were found in demographic and clinical data (Table 4).

**Figure 4:**
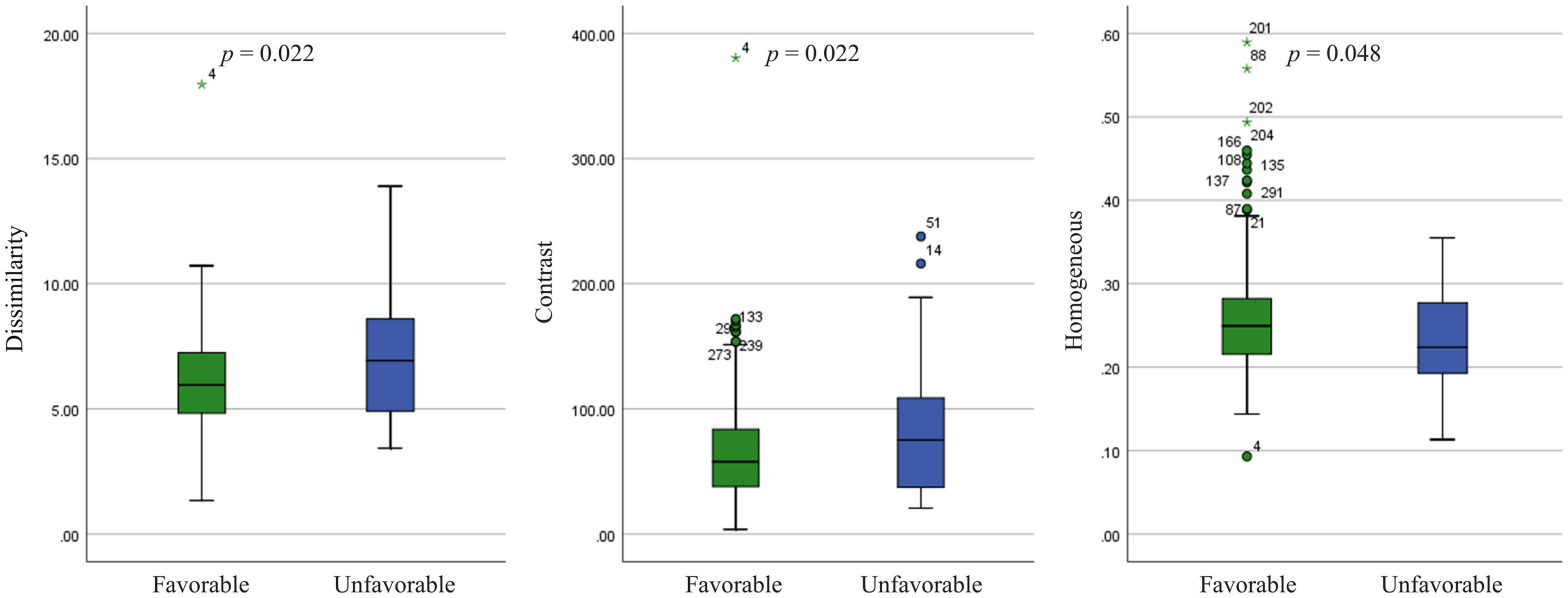
Texture features analysis (*p* < 0.05) in the small artery occlusion type between favorable-outcome and unfavorable-outcome groups.

Three GLCM texture features were significantly different in these two outcome groups. GLCM dissimilarity (*p* = 0.022) and contrast (*p* = 0.022) were higher in the unfavorable-outcome group, whereas homogeneity was higher in the favorable-outcome group (*p* = 0.048) (Figure 4).

In the validation set, the AUC of clinical-texture model was 0.81, and the accuracy, sensitivity and specificity were 0.84, 0.75, and 0.84, respectively. The texture model showed an AUC of 0.74 with accuracy, sensitivity and specificity of 0.84, 0.67, and 0.85, while the AUC of clinical model was 0.64, and the accuracy, sensitivity and specificity were 0.79, 0.50, and 0.81 (Table 3 and Figure 3C).

## Discussion

In this study, we developed new models that could predict 90-day functional outcomes in patients with AIS. Our findings indicated that the second-order texture characteristics reflected the heterogeneity of stroke lesions. The predictive models of LAA and SAO stroke outcomes had moderate sensitivity and specificity. We demonstrated that the texture feature profiles differed between LAA and SAO subtypes.

TA features proved to be efficient in describing the voxel inter-relationships and the gray-level distributions within images, allowing the quantification of the intrinsic heterogeneity invisible to the naked eye. We extracted the second-order features of texture features, which consisted of GLCM, GLRLM, GLSZM, and NGTDM. The GLCM features quantified the relationship between gray levels by counting the pairs with predefined distance and direction that had the same distribution of gray-level values (24). The GLRLM features quantified the length and number of consecutive voxels that had the same gray-level value (25). The GLSZM features quantified the number of connected voxels that shared the same gray-level intensity (26). The NGTDM features quantified the difference between a gray value and the average gray value of its neighbors within the predefined distance (27). The significant differences in second-order texture features might suggest that the spatial inter-relationship between adjacent voxels in patients with AIS having functional independence was different from that in patients with disability.

The pathophysiological mechanisms underlying ischemic stroke termed the ischemic cascade, which consisted of the formation of reactive oxygen species, release of glutamate, accumulation of intracellular calcium, and induction of inflammatory processes. A previous study showed that the texture features based on DWI were closely related to edema after cerebral infarction (28). Electrico-physiological phenomena such as cortical spreading depolarisations with associated energy failure and altered intracellular calcium concentration particularly from cells of the neuromuscular unit resulting into further neuronal cell injury, blood–brain barrier (BBB) break-down and related changes of the microstructures and thereby of the ADC maps and other radionics (29, 30). TA has been useful in examining subtle BBB leakage and inflammatory process after brain ischemia (14, 31). However, the relationship between TA features and pathological changes of stroke is unclear. We speculated that the complex pathophysiological processes of stroke might lead to the microstructural changes, which could be reflected in texture features.

A recent study found that the radiomics signatures based on ADC maps were associated with unfavorable outcomes and served as a risk factor (32). The radiomics features of computed tomography reflected the heterogeneity of stroke infarction and had good performance in predicting patient prognosis (33–35). One recent study also confirmed that a clinical-radiomics nomogram from DWI was a good predictor for ischemic stroke prognosis (36). Our study also demonstrated a positive signal, indicating the ADC-based texture analysis could be a useful tool in predicting stroke prognosis.

In a previous study, several texture features, principally the GLRLM features, differed between patients with AIS undergoing mechanical thrombectomy with good versus bad outcomes (37). Although the findings were partially similar, our study highlighted the differences in texture feature categories by the stroke subtype. We found that adjacent voxel relationships of images had higher dissimilarity and contrast and less homogeneity in unfavorable-outcome patients with SAO than those in favorable-outcome patients, which were diametrically contradictory to previous results (37). These implied that the brain tissue textures on infarction lesions with bad outcomes might be more complex and heterogeneous than those on lesions with good outcomes. Similar results were obtained in previous studies of chronic ischemic stroke (38). Furthermore, we showed that five GLRLM features, seven GLSZM features, and one NGTDM feature were associated with stroke outcomes in LAA, whereas only three GLCM features were associated with stroke outcomes in SAO. One possible explanation could be ascribed to the different pathogenic mechanisms of ischemic stroke. TA has been used to automatically differentiate lacunar syndrome and partial or total anterior circulation stroke based on MRI images (12). The occluded arteries in lacunar infarcts were end arteries, which was in contrast to the large cerebral artery disease; no collaterals were formed with the adjacent vascular territories. The findings of such specific subtypes might support the concept of a different underlying etiologic disease process.

As expected, a larger volume of infarct lesions and increasing stroke severity in terms of the admission NIHSS score were associated with unfavorable outcomes in both the entire cohort and the LAA subtype. This finding was in accordance with the previous literature (39). The reason why no difference was found in age and sex between two groups was probably that the majority of enrolled patients had a good prognosis. The clinical characteristics in SAO did not correlate with the clinical outcomes was likely because the patients with SAO tended to have smaller infarct volumes and mild clinical symptoms.

This study had several limitations. First, the retrospective data might lead to selection bias. Second, the clinical symptoms of patients in this cohort were relatively mild, and a large proportion of patients had a good prognosis. We used deep learning algorithms to tackle the imbalance of data distribution and hence improve the performance of our predictive models. Third, considering our small study population, we did not analyze the textures of the other three subtypes of TOAST. Fourth, we included patients with AIS, however, we did not analyze whether different therapies vary different texture features. Further research is still needed in the future. Fifth, other relevant factors were not accounted for, such as hemorrhagic transformation or white matter hyperintensities, which were previously linked to texture features (40, 41). Future work should contemplate enlarging the sample size, finding TA features relevant to each stroke subtype, and demonstrating the robustness of these results in a prospective randomized multicenter study.

In conclusion, the TA base on ADC maps showed potential value in predicting the prognosis of patients with AIS. TA features differed in LAA and SAO stroke subtypes. Combined with clinical characteristics, TA could be used to improve the efficacy for predicting the functional outcomes in AIS.

## Data availability statement

The original contributions presented in the study are included in the article/Supplementary material, further inquiries can be directed to the corresponding authors.

## Ethics statement

The studies involving human participants were reviewed and approved by the institutional ethics committee of Minhang Hospital. Written informed consent for participation was not required for this study in accordance with the national legislation and the institutional requirements.

## Author contributions

HW: conceptualization. YS: methodology and writing – original draft preparation. BS: formal analysis. JZ: investigation. YZ: project administration. HW: writing – review and editing. YS and HW: funding acquisition. All authors contributed to the article and approved the submitted version.

## Funding

This research was funded by the Natural Science Foundation of Minhang Hospital, Fudan University (2022MHBJ04 and 2022MHPY04), Science and Technology Commission of Minhang District, Shanghai (021MHZ051), and Health Commission of Minhang District, Shanghai (mwyjyx17).

## Conflict of interest

The authors declare that the research was conducted in the absence of any commercial or financial relationships that could be construed as a potential conflict of interest.

## Publisher’s note

All claims expressed in this article are solely those of the authors and do not necessarily represent those of their affiliated organizations, or those of the publisher, the editors and the reviewers. Any product that may be evaluated in this article, or claim that may be made by its manufacturer, is not guaranteed or endorsed by the publisher.

## Supplementary material

The Supplementary material for this article can be found online at: https://www.frontiersin.org/articles/10.3389/fneur.2023.1132318/full#supplementary-material

## Footnotes

^1^ www.itk-snap.org
