# Supplementary material for: Texture analysis of apparent diffusion coefficient maps in predicting the clinical functional outcomes of acute ischemic stroke
Source: Front Neurol. 2023 May 11;14:1132318. doi: 10.3389/fneur.2023.1132318 (PMC10213640; doi:10.3389/fneur.2023.1132318)
Supplement: Supplementary file 1 [file Table_1.DOCX]

**Supplement table 1 Texture features**

| Method | Texture features | Number of features |
| --- | --- | --- |
| GLCM (gray-level co-occurrence matrix) | Energy | 8 |
|  | Contrast |  |
|  | Entropy |  |
|  | Homogeneity |  |
|  | Correlation |  |
|  | Sumaverage |  |
|  | Variance |  |
|  | Dissimilarity |  |
| GLRLM (gray-level run-length matrix) | Short run emphasis (SRE) | 13 |
|  | Long run emphasis (LRE) |  |
|  | Gray-level nonuniformity (GLN) |  |
|  | Run-length nonuniformity (RLN) |  |
|  | Run percentage (RP) |  |
|  | Low gray-level run emphasis (LGRE) |  |
|  | High gray-level run emphasis (HGRE) |  |
|  | Short run low gray-level emphasis (SRLGE) |  |
|  | Short run high gray-level emphasis (SRHGE) |  |
|  | Long run low gray-level emphasis (LRLGE) |  |
|  | Long run high gray-level emphasis (LRHGE) |  |
|  | Gray-level variance (GLV) |  |
|  | Run-length variance (RLV) |  |
| GLSZM (gray-level size zone matrix) | Small zone emphasis (SZE) | 13 |
|  | Large zone emphasis (LZE) |  |
|  | Gray-level nonuniformity (GLN) |  |
|  | Zone-size nonuniformity (ZSN) |  |
|  | Zone percentage (ZP) |  |
|  | Low gray-level zone emphasis (LGZE) |  |
|  | High gray-level zone emphasis (HGZE) |  |
|  | Small zone low gray-level emphasis (SLZGE) |  |
|  | Small zone high gray-level emphasis (SZHGE) |  |
|  | Large zone low gray-level emphasis (LZLGE) |  |
|  | Large zone high gray-level emphasis (LZHGE) |  |
|  | Gray-level variance (GLV) |  |
|  | Zone-size variance (ZSV) |  |
| NGTDM (neighborhood gray-tone difference matrix) | Coarseness | 5 |
|  | Contrast |  |
|  | Busyness |  |
|  | Complexity |  |
|  | Strength |  |
